# Supplementary material for: Developmentally regulated Tcf7l2 splice variants mediate transcriptional repressor functions during eye formation
Source: eLife. 2019 Dec 12;8:e51447. doi: 10.7554/eLife.51447 (PMC6908431; doi:10.7554/eLife.51447)
Supplement: Figure 1—source data 1. — (A) Nucleotide sequence of zebrafish tcf7l2 exon 5 (bold and highlighted) and neighbouring exons. (B) Amino acid sequence of the translated sequence of exons in (A). Amino acids Y128, V161, T172, S175 and L181 numbered. [file elife-51447-fig1-data1.docx]

Zebrafish Tcf7l2 new exon (highlighted) neighbouring exons and translated protein sequence

Nucleotide Sequence

TATCTACAGATGAAATGGCCCCTGCTAGATGTTCAAGCAGGAAGTCTTCAGAGTAGACAAGCACTTAAAGATGCCAGGTCACCTTCTCCAGCACACATCGTTGGGCCCTTCTGCTTGGAATTCCCCGGACAGACTGATCTGAGTCTTCACCAATTACAGTTGTCTAATAAGGTCCCCGTGGTACAGCACCCTCACCATGTGCACCCGCTCACACCTCTGATCACCTACAGCAATGAGCACTTCACGCCTGGGAACCCCCCTCCACATCTACAGGCAGACGTGGACCCCAAAACAG

Protein sequence

^128^YLQMKWPLLDVQAGSLQSRQALKDARSPSPAHIV^161^GPFCLEFPGQT^172^DLS^175^LHQLQL^181^SNKVPVVQHPHHVHPLTPLITYSNEHFTPGNPPPHLQGDVDPKT
