# Supplementary material for: Developmentally regulated Tcf7l2 splice variants mediate transcriptional repressor functions during eye formation
Source: eLife. 2019 Dec 12;8:e51447. doi: 10.7554/eLife.51447 (PMC6908431; doi:10.7554/eLife.51447)
Supplement: Figure 5—source data 1. [file elife-51447-fig5-data1.docx]

| **1** | MPQLNGGGGD | DLGANDEMIS | FKDEGEQEEK | **ISENSSAERD** | **LADVKSSLVN** |
| --- | --- | --- | --- | --- | --- |
| **51** | **ESETNQNSSS** | **DSEAER**RPPP | R**SESFRDKTR** | **ESLEEAAKRQ** | **DGGLFKSPPY** |
| **101** | **PGYPFIMIPD** | **LTSPYLPNGS** | **LSPTARTYLQ** | **MKWPLLDVQA** | **GSLQSR**QALK |
| **151** | **DARSPSPAHI** | **VGPFCLEFPG** | **QTDLSLHQLQ** | **LSNK**VPVVQH | PHHVHPLTPL |
| **201** | ITYSNEHFTP | GNPPPHLQAD | VDPKTGIPRP | PHPPDISPYY | PLSPGTVGQI |
| **251** | PHPLGWLVPQ | QGQPVYPITT | GGFR**HPYPTA** | **LTVNASMSSL** | **LSSR**FPPHMV |
| **301** | PPHHSLHTTG | IPHPAIVTPN | VK**QESSHSDI** | **GSLNSSKHQD** | **AK**KEEEKKKQ |
| **351** | PHIK**KPLNAF** | **MLYMK**EMR**AK** | **VVAECTLKES** | **AAINQILGR**R | WHALSREEQA |
| **401** | KYYELARKER | **QLHMQLYPGW** | **SAR**DNYGKKK | KRKREKQAGE | GNDANTPKKC |
| **451** | RALFGLDQLG | LWCKPCRRKK | KCIRYIQGEG | SCASPPSSDG | SLLESPPSSP |
| **501** | SMISPSPSSK | ESKPQTEQMQ | PLSLTMKPPP | LLSSSQHQHL | SMAPPPPLAL |
| **551** | LDNSGAGK**TS** | **GSAHNGSLDP** | **SDVSSSR**STG | SASSRPISAC | HSHSLLPSSA |
| **601** | PTQPLSLVTK | SID |  |  |  |
